# Study of the BCG Vaccine-Induced Cellular Immune Response in Schoolchildren in Antananarivo, Madagascar

**DOI:** 10.1371/journal.pone.0127590

**Published:** 2015-07-27

**Authors:** Paulo Ranaivomanana, Vaomalala Raharimanga, Patrice M. Dubois, Vincent Richard, Voahangy Rasolofo Razanamparany

**Affiliations:** 1 Mycobacteria Unit, Institut Pasteur de Madagascar, Antananarivo, Madagascar; 2 Epidemiology Unit, Institut Pasteur de Madagascar, Antananarivo, Madagascar; 3 Immunovacc Consulting, Hévillers, Belgium; 4 Epidemiology Unit, Institut Pasteur de Dakar, Dakar, Sénégal; The Catholic University of the Sacred Heart, Rome, ITALY

## Abstract

**Objective:**

Although the Bacillus Calmette-Guérin vaccine (BCG) protects young children against serious forms of TB, protection against pulmonary TB is variable. We assessed BCG vaccine-induced cellular immune responses and determined for how long they could be detected during childhood in Antananarivo, Madagascar.

**Methods:**

We assessed BCG vaccine-induced cellular immune responses by TST and IGRA (in-house ELISPOT assay) using BCG and PPD as stimulation antigen, and compared results between vaccinated and non-vaccinated schoolchildren of two age groups, 6-7 and 13-14 years old.

**Results:**

Three hundred and sixty-three healthy schoolchildren were enrolled. TST was performed on 351 children and IGRA on 142. A high proportion (66%; 229/343) of the children had no TST reactivity (induration size 0 mm). TST-positive responses (≥15 mm) were more prevalent among 13-14 year-old (31.7%) than 6-7 year old (16.5%) children, both in the non-vaccinated (43% vs. 9%, p<0.001) and vaccinated (29% vs. 13%, p=0.002) subgroups. There were no significant differences in TST responses between vaccinated and non-vaccinated children in either of the age groups. The IGRA response to BCG and to PPD stimulation was not significantly different according to BCG vaccination record or to age group. A high rate (15.5%; 22/142) of indeterminate IGRA responses was observed. There was very poor agreement between TST and IGRA-PPD findings (k= 0.08) and between TST and IGRA-BCG findings (k= 0.02)

**Conclusion:**

Analysis of TST and IGRA response to stimulation with BCG and PPD revealed no difference in immune response between BCG-vaccinated and non-vaccinated children; also no decrease of the BCG vaccine-induced cellular immune response over time was observed. We conclude that TST and IGRA have limitations in assessing a role of BCG or tuberculosis-related immunity.

## Introduction

With 9 million cases and 1.5 million deaths in 2013 [1], tuberculosis (TB) remains a major global threat, especially in developing countries. The disease is compounded by the AIDS epidemic and the emergence of multidrug-resistant (MDR) or extensively drug resistant (XDR) TB. The most common presentation of TB disease in adults is pulmonary TB, which is the main source of TB transmission.

In Madagascar, TB is a major public health problem and its incidence remains high. In 2013, the World Health Organization estimated 233 new cases per 100,000 inhabitants [1]. The National TB Control Program (NTCP-Ministry of health) implements the WHO-recommended strategy for detection and treatment of infectious cases. Bacillus Calmette-Guérin (BCG) vaccination at birth is still the main preventive measure against the disease: BCG protects young children against severe forms of TB such as TB meningitis and miliary TB. However, BCG-mediated protection against the adult TB pulmonary form is variable [2,3]. Understanding the mechanisms of action of this vaccine may allow progress towards the restoration of effective host immune protection.

The tuberculin skin test (TST) has been widely used for the detection of mycobacterial infection, and it is also used to assess the induced immune response following BCG vaccination [4,5]. TST elicits delayed hypersensitivity following the intradermal injection of purified protein derivative (PPD), a mixture of antigens, some of which are shared by *Mycobacterium tuberculosis*, *Mycobacterium bovis* BCG, and various non-tuberculous mycobacteria (NTM). TST is cheap and does not require laboratory infrastructure, but also has several limitations. Its execution and reading is dependent on the operator; the interpretation may vary depending on the study population; it requires two visits for each subject; it is not very specific especially in endemic countries; and the sensitivity of the test can be low in immunocompromised subjects [6,7,8]. A recent study conducted with school children aged 6–7 years old in Antananarivo confirmed that the response to the TST was independent of BCG vaccination status [9].

Thus, the TST cannot be considered to be a reliable and fast test for assessing immune protection induced by BCG. Consequently, an alternative is currently recommended: analyzing T-cell IFN-γ (interferon-gamma) expression in response to activation with antigens of the TB complex. Several studies have shown that IFN-γ secretion is critical for controlling *M*. *tuberculosis* infection [10,11]. Moreover, there has been an important advance in recent years with the development of *in vitro* T cell—based IFN-γrelease assays (IGRAs), using more specific *M*. *tuberculosis* antigens, such as ESAT-6 (early secreted antigen target-6) and CFP-10 (culture filtrate protein-10), to assess the BCG-induced cellular immunity and to identify TB infection [12].

A large part of the Malagasy population is administered BCG vaccination at birth. It would be useful to determine, first, the basic immune response conferred by BCG in such vaccinated populations, and secondly, the age until which this response can be detected. Indeed, BCG protection wanes over time.

The aim of this study was to evaluate BCG vaccine-induced cellular immune responses and to determine whether this response can be detected in 6–7 year-old and 13–14 year-old children in Antananarivo.

## Materials and Methods

### Study design

Approval for the study was given by The National Ethics Committee of the Ministry of Health of Madagascar (Authorization N° 030-CE/MINSAN, 04/06/2010).

This study was a cross-sectional study to examine the immune response against mycobacterial antigens (PPD and BCG) in young children who had or had not received BCG vaccination at birth in Antananarivo, Madagascar.

### Participants and Setting

We enrolled participants at two of the most populous areas, Anosibe and Ambohipo, in the capital Antananarivo where the incidence of TB was about 150 cases per 100 000 inhabitants. Healthy children, aged 6–7 years old or 13–14 years old attending two state primary and two state secondary schools (Anosibe, and Ambohipo) were recruited. These children underwent TST and sample collection (whole blood) for the IGRA between June 2010 and October 2010.

### Inclusion

Before inclusion, the study was explained to the children’s parents/guardians. They were also given a letter, which contained a written consent form describing the nature and possible consequences of the study. The parents or guardians of all subjects gave written informed consent in local language at time of recruitment for their children to participate in the study. A standardised questionnaire asking about personal background, clinical data, and the child's vaccination history and TB history was completed. The children’s left and right upper arms were examined for a BCG scar. Children fulfilling the inclusion criteria were identified from the questionnaire.

We included healthy schoolchildren of both sexes, aged 6–7 years and 13–14 years, with and without BCG scars and with parental consent. Sick children and children with a personal or family history of TB were excluded.

### Tuberculin skin test

The Mantoux method was used for TST with 0.1 ml of PPD (Tubersol, Aventis—Pasteur) injected in the dermis; 48–72 h after administration of the TST, the diameter of induration was measured along the long axis of the arm, at home or at school. Children with an induration diameter ≥15 mm were considered to be positive for the TST according to the national guidelines for TB control in Madagascar.

### Blood collection

Peripheral blood samples (7–8 ml) were collected in tubes containing sodium-heparin at school or at home and sent to the Mycobacteria unit at the Pasteur Institute of Madagascar. The study physician recorded the time of blood collection and volume collected at the collection site. Samples were processed immediately on arrival at the laboratory.

### Peripheral blood mononuclear cells (PBMC) isolation

Upon arrival at the laboratory, whole blood was centrifuged at 1200 rpm at room temperature for 5 min. The supernatant (plasma) was collected and stored at -20°C. PBMC were isolated from whole blood by a Ficoll method. Then, the PBMC were collected, washed, counted and cryopreserved at -80°C; after two days they were transferred to liquid nitrogen until the completion of the IGRA.

### IGRA

The IGRA used was an in-house ELISPOT assay: the procedure was modified from that previously described [13,14,15] using PPD and BCG as stimulating antigens. The assay was performed in triplicate for each well condition. Briefly, PBMC were thawed and counted, then placed in 96-well plates with well membranes of polyvinylidene fluoride (PVDF Millipore): the plates had already been coated for 2 hours with 100μl/well of mouse anti-human IFN-γ monoclonal antibody (mAb 1-D1K, cat: 3420-3-1000, MabTech) at 10 μg/ml, washed in phosphate-buffered saline (PBS), and blocked with R10 medium. Cells were plated at 10^5^ cells/well, and incubated for 18 hours (overnight) at 37°C with 10μg/ml PPD (Tubersol, Aventis Pasteur) and with 1/100 diluted BCG solution (viable BCG Vaccine, Serum Institute of India LTD, 1–33 x 10^5^ CFU/ml) as antigens based on the optimal responsiveness detected in prior titration studies; RPMI 1640 medium and 5μg/ml PHA (cat: L-2769; Sigma) were used as a negative and positive controls, respectively. For the detection of IFN-γ plates were incubated for 4 hours at 37°C with 100 μL per well of biotinylated antihuman IFN-γ monoclonal antibody (Anti-human Interferon-γ, mAb 7-B6-1-Biotin, cat: 340-6-250, batch: 39.1, MabTech) at 1 mg/mL, and then for 2 hours at 37°C with 100μL/well of a 1 mg/mL solution of alkaline phosphatase-conjugated streptavidin (AP Conjugate Substrate Kit, cat: 170–6432, BIO-RAD). After several washes, plates were incubated for 3 to 5 minutes at room temperature with 100 mL per well of 1-Step substrate color-development (AP Conjugate Substrate Kit, cat: #170–6432, BIO-RAD) to develop spots. Once spot-forming cells (SFCs) were clearly visible in the positive control (PHA), tap water was added all wells to stop the reaction. The Elispot plates were then dried, and SFCs were counted by two operators, blind to the nature of the samples, using a dissecting microscope. One SFC corresponds to one IFN-γ secreting cell. The mean number of SFCs counted by the two operators for each well was calculated. The results are expressed as SFC counts/10^5^ cells. The result was considered indeterminate if, independently for each subject, there was a lower response to mitogen (PHA) than to antigens (PPD or BCG) and also a higher nil response (RPMI) than to these antigens. The ELISPOT result for each subject was analysed by a non-parametric statistical test: DFR(2x) (distribution free resampling) [14,15], available online at http://scharp.org/zoe/runDFR/ [16]. This test allows a qualitative IFN-γ response to be obtained as positive or negative after P-value calculation, for each subject and for each antigen independently (PPD and BCG). It also allows for a false positive rate lower than 1%.

### Data management and statistical analysis

Information (clinical data, BCG vaccination status, age, gender, TST result) for each subject were entered into an ACCESS database. The presence of BCG scar was considered to be a validated proof of vaccination.

The data were analyzed to study the variation of the TST response and the variation of IGRA response according to the BCG vaccination record and to the age group. The proportions and distributions of the results were compared between the different groups of children with the *Chi-squared* test or Fisher’s exact test when expected group cell sizes were smaller than five. A *P-value* <0.05 was considered statistically significant and 95% confidence intervals (95%CI) were constructed around estimates. Agreement between TST and IGRA results was assessed with the kappa coefficient statistic test [17].

## Results

### Characteristics of study population

**Fig 1:**
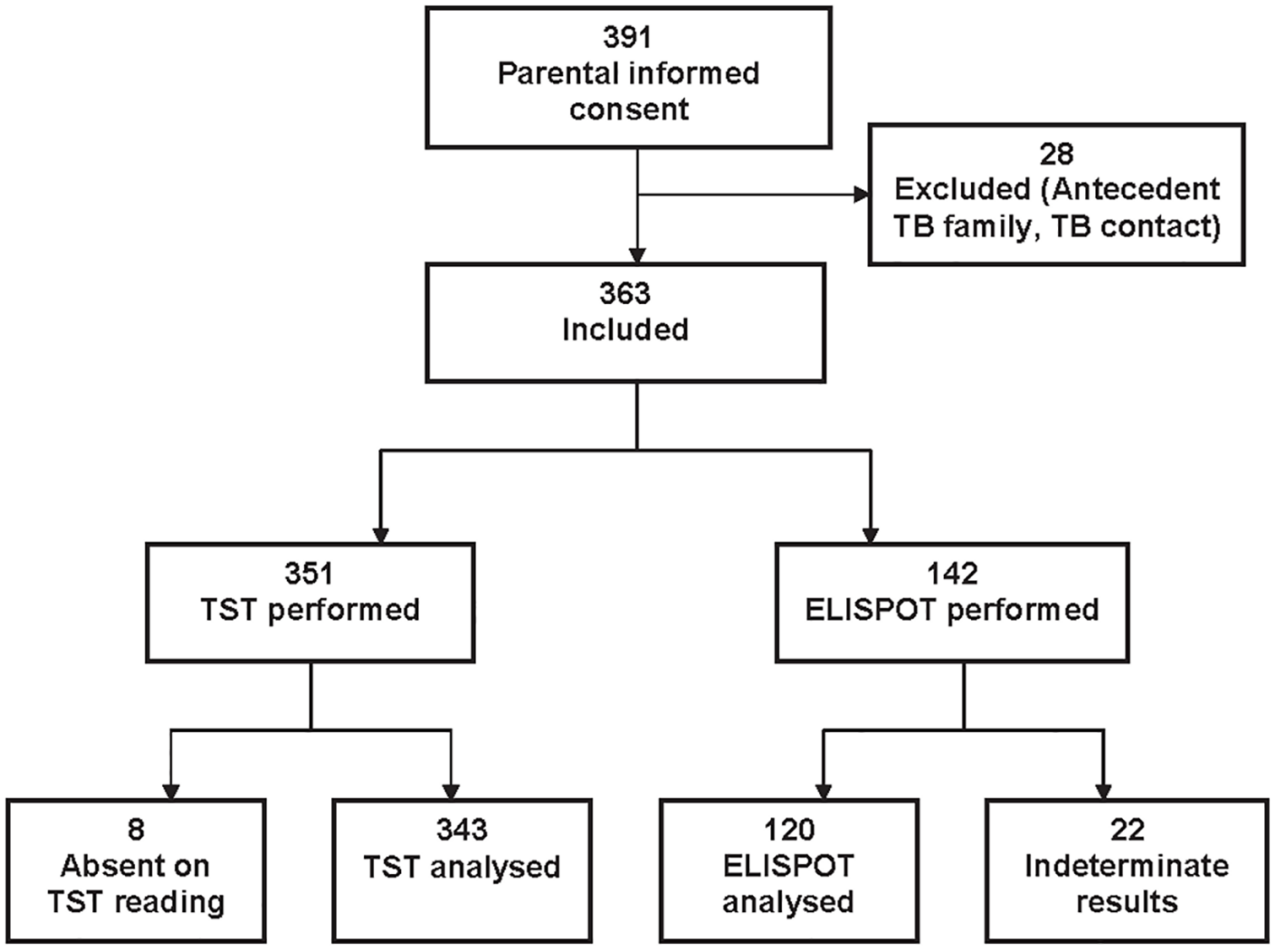
Flow chart of the study on BCG vaccine immune response in children, Antananarivo, 2010–2011.

After obtaining all the necessary arrangements for the study, the study physician received parental consent for 391 schoolchildren in four state schools (two primary and two secondary) in Antananarivo. After application of the inclusion and exclusion criteria, 363 children were included in the study (Fig 1). The distribution and classification of children according to age group (6–7 and 13–14 years old) and presence of a BCG scar is given in Table 1.

**Table 1:** General characteristics of the study population.

	Age group (years old) N (%)		
**Gender**	**6–7**	**13–14**	**N = 343**
Male	100 (48.3)	72 (52.9)	172 (50.0)
Female	107 (51.7)	64 (47.1)	171 (50.0)
**Presence of BCG scar**			
No	45 (21.7)	49 (36.0)	94 (27.4)
Yes	162 (78.3)	87 (64.0)	249 (72.6)

TST was performed on 351 of the 363 children and 343 were included in the final analysis. IGRA by ELISPOT was performed in 142 children of whom 120 were included in the final analysis (Fig 1). Twenty-two children with TB antecedent, 15 (6–7 years old) and 7 (13–14 years old), were excluded.

### Results of tuberculin skin test

The TST was finally analysed in a total of 343 children (S1 Table). There was no significant difference in TST response according to sex or school. No TB symptoms were detected by chest X-ray in any of the children with a positive TST response.

Several studies reported a high proportion of children with no measurable TST reaction [9, 18,19]. For 66.8% (229/343) of the children, TST reactivity was undetectable, with an induration size of 0mm (Table 2). The proportion of children with no TST reactivity was significantly higher in the 6–7 year-old group (77.7%; 161/207) than in the 13–14 year-old group (50%; 68/136) (p<0.001) (Table 2). A large proportion of children in both age groups (83.5% of the 6–7 year-olds and 68.3% of the 13–14 year-olds) had negative TST results (<15mm; Table 3). We compared the 6–7 to the 13–14 year-olds: regardless of the BCG vaccination status, significantly more children in the 13–14 year-old group (31.7%) than in the 6–7 year-old group (16.5%) had positive TST responses (p = <0.001, Table 3). These differences in TST responses according to age were observed for both BCG vaccination status groups (non-vaccinated and vaccinated groups) (Table 4).

**Table 2:** Tuberculin skin test reactivity scored as presence (>0mm) or absence (0mm) of induration according to age group (years old).

		TST (mm)			
Age group (years old)	Total	0	>0	P-value	OR [95% CI]
**6–7**	207	161 (77.7)	46 (22.3)	<0.001*	1
**13–14**	141	68 (50.0)	68 (50.0)		3.5 [2.2–5.6]
**Total**	343	229 (66.8)	114 (33.2)		

TST: Tuberculin skin test; OR [95% CI]: odds ratio [95% confidence interval]

* Comparison of TST results between the two age groups.

**Table 3:** Overall distribution of tuberculin skin test results (by induration size).

		TST (mm)			
Age group (years old)	Total	[0–14]	≥15	P-value	OR [95% CI]
**6–7**	207	182 (83.5)	25 (16.5)	<0.001*	1
**13–14**	136	90 (68.3)	46 (31.7)		3.7 [2.1–6.7]
**Total**	343	272 (79.3)	71 (20.7)		

TST: Tuberculin skin test; OR [95% CI]: odds ratio [95% confidence interval]

* Comparison of TST results between the two age groups.

**Table 4:** Distribution of tuberculin skin test results (by range of induration size) according to the age group within the two BCG scar record groups.

		TST (mm)			
BCG scar record	Age group (years old)	[0–14]	≥15	P-value	OR [95% CI]
**NV** n = 94	**6–7**	41 (91.1)	4 (8.9)	<0.001*	1
	**13–14**	28 (57.1)	21 (42.9)		7.7 [2.2–30.0]
**V** n = 249	**6–7**	141 (87.0)	21 (13.0)	0.002*	1
	**13–14**	62 (71.3)	25 (28.7)		2.7 [1.3–5.5]
**Total**	N = 343	272	71		

TST: Tuberculin skin test; NV: non-vaccinated; V: vaccinated; OR [95% CI]: odds ratio [95% confidence interval]

*Comparison of TST results between the two age groups.

However, there was no difference in TST response between BCG vaccinated and non-vaccinated children within each age group (p = 0.5 for 6–7 and p = 0.09 for 13–14 year-olds) (Table 5).

**Table 5:** Distribution of tuberculin skin test results (by induration size) according to BCG scar status within the two age groups.

		TST (mm)			
Age group (years old)	BCG scar status	[0–14]	≥15	P-value	OR [95% CI]
**6–7** n = 207	**NV** n = 45	41 (91.1)	4 (8.9)	0.5*	1
	**V** n = 162	141 (87.0)	21 (13.0)		1.5 [0.5–5.6]
**13–14** n = 136	**NV** n = 49	28 (57.1)	21(42.9)	0.09*	1
	**V** n = 87	62 (71.3)	25 (28.7)		0.5 [0.2–1.2]
**Total**	N = 343	272	71		

TST: Tuberculin skin test; NV: non-vaccinated; V: vaccinated; OR [95% CI]: odds ratio [95% confidence interval]

*Comparison of TST results between the vaccinated and the non-vaccinated groups of children.

### IGRAs responses according to BCG vaccination status (scar record) and to age group

The ELISPOT assay was performed on PBMC samples from 142 children (S1 Table). Twenty-two (15.4%) gave indeterminate responses, so 120 results were included in the final analysis (Fig 1). We tested for a difference in IFN-γ response according to the BCG vaccination status within the two age groups of children (Table 6): following stimulation with BCG, there was no significant difference in the IFN-γ response between non-vaccinated and vaccinated children in either age group (p>0.05). Similar results were observed following stimulation with PPD (Table 7).

**Table 6:** Distribution of IGRA response (to BCG stimulation) according to BCG scar record within the two age groups.

Age group (years old)	IGRA	NV	V	Total	P-value	OR [95% CI]
**6–7**	**Neg**	2 (22.2)	27 (57.4)	29 (51.8)	0.07*	1
	**Pos**	7 (77.8)	20 (42.6)	27 (48.2)		0.2 [0.03–1.3]
**13–14**	**Neg**	13 (59.1)	24 (57.1)	37 (57.8)	0.9*	1
	**Pos**	9 (40.9)	18 (42.9)	27 (42.2)		1.1 [0.3–3.5]
	***P-value***	0.1	1	0.5		

IGRA: interferon-gamma release assay; TST: Tuberculin skin test; NV: non-vaccinated; V: vaccinated; BCG: Bacille Calmette and Guérin; Neg: negative; Pos: positive; OR [95% CI]: odds ratio [95% confidence interval]

*Comparison of IGRA results between the vaccinated and the non-vaccinated groups of children.

**Table 7:** Distribution of IGRA response (to PPD stimulation) according to BCG scar record within the two age groups.

Age group (years old)	IGRA	NV	V	Total	P-value	OR[95% CI]
**6–7**	**Neg**	3(33.3)	30 (63.8)	33 (58.9)	0.09*	1
	**Pos**	6 (66.7)	17 (36.2)	23 (41.1)		0.3 [0.05–1.5]
**13–14**	**Neg**	11 (50.0)	29 (69.0)	40 (62.5)	0.1*	1
	**Pos**	11 (50.0)	13 (31.0)	24 (37.5)		0.5 [0.1–1.5]
	***P-value***	0.4	0.6	0.7		

IGRA: interferon-gamma release assay; TST: Tuberculin skin test; NV: non-vaccinated; V: vaccinated; PPD: Purified Protein Derivative; Neg: negative; Pos: positive; OR [95% CI]: odds ratio [95% confidence interval]

*Comparison of IGRA results between the vaccinated and the non-vaccinated groups of children.

We then tested for differences in the IFN-γ response according to age group. There was no difference in IFN-γ responses between the vaccinated 6–7 year-old and the vaccinated 13–14 year-olds, following stimulation with either BCG (p = 1, Table 6) or PPD (p = 0.6, Table 7). However, the proportion of IGRA-positive responses to PPD and BCG was higher among non-vaccinated 6–7 year-olds than non-vaccinated 13–14 year-olds, although the difference was not statistically significant (p = 0.1 for PPD, Table 6 and p = 0.4 for BCG, Table 7).

### Agreement between TST and IGRA

Agreement between the TST and IGRA results could be assessed for 120 children (Tables 8 and 9). There was a very poor agreement between TST and IGRA-PPD results (*k* = 0.08) and between TST and IGRA-BCG results (*k* = 0.02). Both IGRA-PPD and IGRA-BCG were positive in only 13 (46%) of the 28 children with positive TST results.

**Table 8:** Agreement between IGRA (to PPD stimulation) and TST results.

		IGRA					
		Pos	Neg	Total	*k*	P-value	OR [95% CI]
**TST**	**Pos**	13	15	28 (23.3)	0.08	0.36	1
	**Neg**	34	58	92 (76.7)			1.4 [0.5–3.7]
	**Total**	47 (39.2)	73 (60.8)	120			

IGRA: interferon-gamma release assay; TST: Tuberculin skin test; Neg: negative; Pos: positive;

*k*: kappa coefficient; OR [95% CI]: odds ratio [95% confidence interval].

**Table 9:** Agreement between IGRA (to BCG stimulation) and TST results.

		IGRA					
		Pos	Neg	Total	*k*	P-value	OR [95% CI]
**TST**	**Pos**	13	15	28 (23.3)	0.02	0.86	1
	**Neg**	41	51	92 (76.7)			1.08 [0.4–2.7]
	**Total**	54 (45.0)	66 (55.0)	**120**			

IGRA: interferon-gamma release assay; TST: Tuberculin skin test; Neg: negative; Pos: positive;

*k*: coefficient kappa.

## Discussion

We analyzed the BCG-induced cellular immune response by TST and IGRA in vaccinated and not vaccinated children of two different age groups: 6–7 years old, and 13–14 years old. All the children were healthy, with no history of TB and no known TB contact in their household.

No induration was visible following TST (induration diameter of 0mm) for 66% of the children. This confirms previous findings for 6–7 year-old, first-year, Malagasy schoolchildren [9]. The proportion of no reactivity to TST was higher in this younger group than the older group (13–14 years old), and a high proportion of children in both age groups had a negative TST result (86.7% and 65.5% for 6–7 and 13–14 year-old groups, respectively; Table 2). This absence of TST reactivity has been decribed in numerous studies of children: 79.5% in 6–7 year-olds in Madagascar [9], 78.7% for 8–14 year-olds in Iran [18], and 76.3% for 3–19 year-olds in Lebanon [19]. This may be a consequence of BCG vaccination being given at birth: maturation of the immune system by age 3 months may allow a much better immunizing effect for later vaccination, as reported previously [20]. Note that TST is less reliable among children with malnutrition and stunted children [21], but these factors were not checked in this study.

The proportion of positive TST responses was significantly higher among children aged 13–14 than those aged 6–7 (p<0.001). However, TST reactivity did not differ significantly between vaccinated and non-vaccinated children. In other words, there was no evident influence of BCG vaccination on the TST results within the two age groups. Therefore, we suggest that differences in TST responses between the two age groups are not related to BCG vaccination, but reflect greater exposure to TB in the older group of children. By age 13, particularly in TB endemic countries like Madagascar, children would be expected to have had numerous contacts with TB patients outside the family, and this may induce TST reactivity. Moreover, children are also at high risk of exposure to non-tuberculosis mycobacteria (NTM) that may lead to TST positive results [22]. Our results are consistent with those of a study in an Amazonian population where the odds of TST reactivity increased by 7.4% per year of age and doubled for every 10-year age interval [23]. However, this previous study also found in multivariate analysis (controlling for sex, age, and TB history) that previous BCG vaccination increased the chance of a positive TST reaction nine fold, but this difference of TST reactivity disappeared when the history of vaccination was taken alone as an independent variable [23]. Thus, further work is needed to clarify the real influence of BCG vaccination on TST results and the influence of numerous factors such as age, risk of TB infection, nutritional status, co-morbidity, HIV status, geographical setting and population heterogeneity. The number of children with negative or absent TST reactivity, regardless of BCG vaccination history, was very high in our study; consequently, because TST is relatively simple to perform, it may be useful for detecting TB infection among the children's contacts as previously suggested [24]. However, TST has several drawbacks, and in particular that two visits are needed. Numerous previous studies reveal that TST reading and interpretation are very subjective and dependent on many factors including the prevailing TB prevalence and BCG vaccination coverage rate. The most important weakness of this test is its low specificity, a consequence of PPD antigen being also present in BCG vaccine strains and in some NTM [25] significantly affecting interpretation of the results.

The immune response against TB is characterized by Th1 cellular immunity, and particularly IFN-γ production. IFN-γ has been described in several studies as one of the key molecules for controlling TB infection. It is possible that IFN-γ serves as a surrogate marker of TB infection [26], so we used this cytokine as a marker of protection against TB infection. Unlike the results of the TST, there was no difference in the IFN-γ response (assessed by IGRA) between the two age groups, either for non-vaccinated or vaccinated children. This indicates that the IGRA-BCG/PPD response is not dependent on age. However other variables like the amount of PPD or BCG used may influence T-cell reactivity. These should be further investigated.

There was also no difference in IGRA response between non-vaccinated and vaccinated children within each of the two age groups. IFN-γ may be only one component of the protective response against TB [27] and the suitability of IFN-γ as a marker of protective immunity (for acquired immunity or vaccine immunity) against TB needs to be confirmed [28]. BCG-induced immune responses have been shown to correlate with cytokine production more generally, and therefore it would be valuable to assess expression of, for example, TNF-α, IL-12, IL-5, IL-10, IL-13, IL-17, MIP-1α, MIP-1β [29], and IP-10 (IFN-γ-inducible protein 10, also known as CXCL10). IP-10 has been described as a promising biomarker of TB [30] and useful for detecting TB infection, especially in children [31,32]. Furthermore, although CD4 T cells play an essential role in controlling *M*. *tuberculosis* infection, the role of other effector cells, such as CD8 T cells, NK cells and γδT cells, should be considered [28].

Our results raise, again, questions about the effectiveness of the BCG vaccine because we did not find any significant difference in BCG-induced immune response between vaccinated and non-vaccinated children. BCG vaccination can be dependent on several factors, including the type of BCG strain used, the methods of culture and conservation, and factors related to the host like the ability to respond to BCG [33]. It is not clearly established whether giving BCG vaccination at birth or to older children is more effective for inducing robust protection. A study of Ugandan infants reported that vaccination at birth was associated with a greater induction and proliferation of effector T-cells (CD4+ and CD8+) expressing IFN-γ, TNF-α, IL-2 and perforin than following vaccination at 6 weeks of age [34]. However, a conflicting result had previously been reported in a South African study where frequencies of BCG-specific polyfunctional CD4+ T cells co-expressing IFN-γ, TNF-α and IL-2 were higher among infants who received BCG vaccination at 10 weeks of age than at birth [35]. It has been suggested that the differences between the results of these two studies were consequences of geographical and population heterogeneity [34]. Indeed, BCG vaccine effectors are believed to involve T-cells with distinct patterns of cytokine production, and particularly a Th1-immune response activation profile [36,37,38]. However, it has not been demonstrated whether or not these responses truly correlate with protection against TB. A cohort study was conducted in South Africa to address this issue: it reported that after neonatal BCG vaccination, there was no difference in the magnitude and profile of cytokine expression of BCG-specific CD4+ and CD8+ T cells between infants supposed to be protected (infants who did not develop TB disease despite exposure to TB in the household) and those supposed to be unprotected (infants who had developed culture-positive TB) after 2 years follow up [39]. Thus, further work is needed to identify biomarkers specific for, or correlating with, TB protection that could be used for, among other purposes, assessing the efficacy of novel TB vaccines in clinical trials.

In conclusion, IFN-γ responses did not allow discrimination between vaccinated and non-vaccinated children, and provided no evidence of any drop in the immune protection induced by BCG over time.

Finally, we observed discordance between the results of TST and IGRA-PPD *(k* = 0.08) and between TST and IGRA-BCG (*k* = 0.02). A recent study similarly reported very poor agreement between TST and PPD-IGRA with *k* = 0.004 [40]. In addition to our finding that BCG vaccination has little influence on the TST response, numerous previous studies have raised questions about the performance of IGRAs [41,42,43,44,45,46,47,48,49]. However, one problem with assessing the performance of the IGRA, as in our study, is the absence of a gold standard test. We observed a high rate of indeterminate responses with the Elispot test (15.4%), similar to what has been reported in numerous previous studies [41,50,51,52,53]. An indeterminate response can be associated with a number of factors including young age, immunosuppresion status [54] and sample handling [55,56].

The limitations of this study include the small numbers of children included, and particularly the small number of non-vaccinated children which was a consequence of systematic BCG vaccination at birth in the country. The study was also restricted to two urban areas in Antananarivo. It would be interesting to repeat this type of study in other settings, not only in Antananarivo but also in other cities and in rural areas. Also, we did not also have information for numerous factors that could bias our results, including in particular malnutrition and HIV status.

## Conclusion

Overall, using TST and IGRA (IGRA-PPD and IGRA-BCG) to evaluate TB immune responses we did not find differences between vaccinated and non-vaccinated children or between the two age groups (6–7 and 13–14 years old). We found substantial discordance between TST and IGRA results. Nevertheless, TST is still used worldwide as a standard test to assess TB infection, and when this study was initiated IGRA assays were considered to be potential replacements for the TST to assess the BCG-induced cellular immune response. However, many unknowns and the lack of a gold standard test are such that the true performance of these tests in high burden countries remains unclear. IFN-γ is not the only component of immune protection induced by BCG vaccine, and other TB immune components should be assessed as the BCG vaccine-induced immune response may be complex. Using multivariate analysis and multiplex assays to investigate a broader cytokine/chemokine profile, and assessing the role of other effector cells (CD8 T cells, NK cells and γδT cells) using a large panel of *M*. *tuberculosis* specific antigens, may be suitable approaches to evaluating BCG-induced immune responses; these approaches require further investigation. Indeed, the identification of biomarkers of protection would be very helpful in research and clinical trials for the development of new candidate TB vaccines.

## Supporting Information

S1 Table Dataset of the participants. **Sex:** M: male; F: female. **BCG vaccine:** 0: Not vaccinated; 1: vaccinated with proof (with vaccination book); 2: vaccinated without proof (no vaccination book); 9: unknown. **TST result:** TST induration diameter (mm). **Blood sample N°:** Identification number of the patient blood sample. **Elispot/BCG1:** Mean of spot count (by two operators) in the first BCG stimulated well. **Elispot/BCG2:** Mean of spot count (by two operators) in the second BCG stimulated well. **Elispot/BCG3:** Mean of spot count (by two operators) in the third BCG stimulated well. **Elispot/PPD1:** Mean of spot count (by two operators) in the first PPD stimulated well. **Elispot/PPD2:** Mean of spot count (by two operators) in the second PPD stimulated well. **Elispot/PPD3:** Mean of spot count (by two operators) in the third PPD stimulated well. **Elispot/RPMI1:** Mean of spot count (by two operators) in the first control well. **Elispot/RPMI2:** Mean of spot count (by two operators) in the second control well. **Elispot/RPMI3:** Mean of spot count (by two operators) in the third control well. **a1 (BCG):** Ajusted value of the column I (Elispot/BCG1). **a2 (BCG):** Ajusted value of the column J (Elispot/BCG2). **a3 (BCG):** Ajusted value of the column K (Elispot/BCG3). **c1:** Ajusted value of the column O (Elispot/RPMI1). **c2:** Ajusted value of the column P (Elispot/RPMI2). **c3:** Ajusted value of the column Q (Elispot/RPMI3). **DFR(2x) adjp(BCG):** P value obtained with the DFR test (with BCG antigen). **DFR(2x) response(BCG):** 0 = p value >0.05 (negative test with BCG antigen); 1 = p value <0.05 (positive test with BCG antigen). **id2:** Identification number of the patient blood sample. **id (PPD):** Identification number of the patient blood sample. **a1(PPD):** Ajusted value of the column L (Elispot/PPD1). **a2(PPD):** Ajusted value of the column M (Elispot/PPD2). **a3(PPD):** Ajusted value of the column N (Elispot/PPD3). **c1:** Ajusted value of the column O (Elispot/RPMI1). **c2:** Ajusted value of the column P (Elispot/RPMI2). **c3:** Ajusted value of the column Q (Elispot/RPMI3). **DFR(2x) adjp(PPD):** P value obtained with the DFR test (with PPD antigen). **DFR(2x) response(PPD):** 0 = p value >0.05 (negative test with PPD antigen); 1 = p value <0.05 (positive test with PPD antigen).
(XLSX)
